# Characterization of broad-host-range *Salmonella* phage GSP006 and its efficacy in controlling *Salmonella* Pullorum contamination in poultry feed and drinking water

**DOI:** 10.1186/s12896-026-01100-w

**Published:** 2026-01-15

**Authors:** Shenyu Pang, Hongyang Zhang, Xincong Liu, Jilai Wang, Shunyuan Pan, Xiangyu Kong, Jun Song, Dongyang Gao

**Affiliations:** 1 College of Animal Science and Veterinary Medicine, Heilongjiang Bayi Agricultural University, Daqing, 163319 China; 2 China Key Laboratory of Bovine Disease Control in Northeast China, Ministry of Agriculture and Rural affairs, Daqing, 163319 China; 3 Heilongjiang Provincial Key Laboratory of Prevention and Control of Bovine Diseases, Daqing, 163319 China

**Keywords:** Phage, *Salmonella* Pullorum, Poultry, Horizontal transmission

## Abstract

**Background:**

*Salmonella enterica* subsp. *enterica* serovar Pullorum (*Salmonella* Pullorum) is the major pathogen that is harmful to the poultry industry in developing countries. It causes acute systemic and severe gastrointestinal diseases in chicks, resulting in high mortality. As a natural alternative to conventional antimicrobial agents, phage therapy is increasingly recognized as highly effective and promising for the control of multidrug-resistant bacterial infections, including salmonellosis caused by *Salmonella*.

**Results:**

In this study, a broad-host-range phage, GSP006, targeting *Salmonella* Pullorum was isolated from poultry farm wastewater. It exhibited lytic activity against *Salmonella* Pullorum, *Salmonella* Enteritidis, *Salmonella* Typhimurium and other *Salmonella* serotypes. Genomic analysis revealed that GSP006 possesses a double-stranded DNA (dsDNA) genome of 42,165 bp with a G + C content of 50%. Phylogenetic analysis based on the terminase large subunit confirmed that GSP006 belongs to the genus *Jerseyvirus*. Biological characterization showed that phage GSP006 was stable to heat (4 °C to 60 °C) and pH (pH 3 to pH 11). A one-step growth curve analysis revealed a short latent period of approximately 20 min, followed by a pronounced lysis phase. In vitro experiments, phage GSP006 was able to inhibit the bacterium for more than 6 h at 37 °C under different infection multiplicities. Furthermore, in antimicrobial assays using poultry feed and drinking water matrices, GSP006 (at MOIs of 100, 1,000, and 10,000) significantly reduced the load of *Salmonella* Pullorum. These results suggest that phage GSP006 may serve as a preventive measure against the horizontal transmission of *Salmonella* Pullorum.

**Conclusions:**

The broad-host-range phage GSP006 demonstrates excellent environmental stability and significantly reduces *Salmonella* Pullorum levels in poultry feed and drinking water under controlled laboratory conditions. This study establishes a foundation for its potential development as an effective biocontrol agent to reduce the horizontal transmission of *Salmonella* Pullorum.

**Supplementary Information:**

The online version contains supplementary material available at 10.1186/s12896-026-01100-w.

## Introduction

*Salmonella* is a common foodborne pathogen belonging to the family *Enterobacteriaceae*, which is widely distributed in nature, especially in poultry, livestock and their products [1, 2]. In the poultry industry, *Salmonella* Pullorum causes acute systemic and severe gastrointestinal diseases in chicks, with high lethality rates, making it one of the most serious pathogens affecting this sector [3]. This pathogen can be transmitted vertically to chicks through infected eggs or horizontally through direct contact with infected/dead chicks, their feces, or contaminated environments [4]. While *Salmonella* Pullorum rarely causes severe clinical signs in adult chickens [5] and has been successfully controlled in developed countries, it remains a major threat in many developing regions with emerging intensive poultry production systems [4].

The overuse of antibiotics not only reduces efficacy but also pollutes the environment and increases public health risks [6]. In recent years, many countries have banned the use of antibiotics as growth promoters in animal feed [7, 8]. Along with increasing public awareness of sustainable development has increased the demand for eco-friendly biological alternatives. This has brought phage therapy back into the public eye as a hot topic [9]. Phages (bacteriophage) are viruses that infect and kill bacteria. They are the most ubiquitous organisms on Earth [10, 11]. As natural antimicrobial agents, phages possess several advantages over conventional antibiotics, including the ability to self-replicate at the site of infection, host specificity, low toxicity, and no cross-resistance with antibiotics [12, 13]. In recent years, there has been a dramatic increase in phage therapy research, especially in the United States (U.S). There is already a precedent for the U.S. Food and Drug Administration (FDA) to approve phage therapy for clinical trials, and the European Union and other countries have relaxed their regulations on phage therapy [14]. Studies have demonstrated that phages exhibit significant potential in controlling bacterial diseases in poultry. Through targeted preventive or therapeutic interventions during farming practices, phages can substantially mitigate the adverse effects of *Salmonella* on animal health and production performance [15–18]. However, due to the diverse serotypes of *Salmonella* and their propensity to develop resistance under phage pressure, future efforts should focus on isolating additional phage resources and systematically elucidating their biological characteristics and genomic features. This will facilitate the establishment of a more comprehensive phage library and promote the effective application of phage-based strategies for *Salmonella* control in poultry.

In this study, a broad-host-range *Salmonella* phage, GSP006, was successfully isolated and identified. Its biological characteristics and genomic features were systematically characterized. To evaluate the efficacy of phage GSP006 in controlling the horizontal transmission of *Salmonella* Pullorum, simulated contamination experiments of poultry feed and drinking water were performed. Phage GSP006 was introduced at varying concentrations into the contaminated matrices. The results demonstrated a significant reduction in *Salmonella* Pullorum counts in the treatment groups compared to the control group. These findings not only confirm the potential of phage GSP006 as a biocontrol agent, but also provide key experimental evidence and theoretical support for developing novel strategies to mitigate the horizontal transmission of *Salmonella* Pullorum in chicks.

## Materials and methods

### Bacterial strains and phage in this study

Phage GSP006 was isolated from farm wastewater in Daqing, China. The *Salmonella* Pullorum SaP001 was used as the trapping host for phage isolation. The bacterial strains used in the host range assay are listed in Supplementary Tables 1 and 2. Strains were cultured in LB broth at 37 °C for 16 h with constant shaking, and bacteria were stored frozen at -80 °C with 25% glycerol until subsequent analysis.

### Phage isolation and purification

Phage isolation and purification were performed using a double-layer plate method as described previously [19]. Briefly, 5 mL of wastewater sample was centrifuged at 6,000 *× g* for 5 min at 4 °C, and the supernatant was filtered through a 0.22 μm filter. Then, 3 mL of the filtrate was combined with 2 mL of host culture and 3 mL of LB medium, followed by incubation at 37 °C for 6 h. The cultures were centrifuged at 10,000 × *g* for 5 min at 4 °C, and the resulting supernatant was filtered through a 0.22 μm filter. Next, 100 µL of the filtrate was mixed with 100 µL of the indicator strain and 5 mL of melted LB soft agar (0.7% agar), and the mixture was overlaid onto an LB agar plate (1.5% agar). After 6 h of incubation at 37 °C, a single clear plaque was picked from the bilayer plate using a sterile needle tip and resuspended in SM buffer (100 mM NaCl, 10 mM MgSO_4_, 50 mM Tris-HCl, pH 7.5). The resuspended phage was serially diluted in SM buffer and subjected to three rounds of purification via the double-layer agar method.

### Determination of host range and efficiency of plating

The host range of phage GSP006 was determined by the efficiency of plating (EOP) as described previously [20]. In brief, freshly propagated phage GSP006 (10^9^ PFU/mL) was serially diluted 10-fold (10^− 3^ to 10^− 9^) in SM buffer. Aliquots (10 µL) of each dilution were added dropwise to bacterial lawns of test strains. The EOP was defined as the ratio of the phage titer on the test strain to the phage titer on the host strain.

### Morphology analysis by transmission electron microscopy

Phage morphology was examined by transmission electron microscopy (TEM) following the method described by Wang et al. [21]. Briefly, a droplet of the phage lysate was applied to a carbon-coated copper grid (200 mesh; Beijing Zhongjingkeyi Technology Co., Ltd.). The grid was then negatively stained with 2% (w/v) phosphotungstic acid (pH 6.5). The samples were observed and imaged using a Hitachi H-7650 transmission electron microscope (Hitachi, Tokyo, Japan) operating at an acceleration voltage of 100 kV.

### Determination of optimal multiplicity of infection (MOI)

The MOI was tested following previously described procedures with some modifications [22]. Briefly, phage lysate was added to mid-logarithmic phase cultures of *Salmonella* Pullorum SaP001 (approximately 10^7^ CFU/mL) to achieve target MOIs of 100, 10, 1, 0.1, 0.01, 0.001, and 0.0001. The cultures were then incubated at 37 °C with shaking for 4 h. After incubation, the samples were centrifuged at 10,000 × *g* for 1 min at 4 °C. The phage titers in the resulting supernatants were determined using the double-layer agar plate method. The MOI that produced the highest phage titer was designated as the optimal MOI.

### One-step growth curve

One-step growth curves were performed as previously described with modifications [23]. Briefly, the bacterial strain SaP001 was infected with phage at an MOI of 0.1 and incubated at 37 °C for 15 min. The mixture was then centrifuged at 10,000×*g* for 1 min at 4 °C. The pellet was washed three times with LB broth to remove unadsorbed phages and finally resuspended in an equal volume of LB broth. The resuspended mixture was immediately incubated in a shaker at 37 °C with oscillation at 160 rpm. Samples (200 µL) were collected every 10 min (up to 150 min) and centrifuged at 10,000×*g* for 1 min at 4 °C. The phage titer in the supernatant was determined at each time point using the double-layer agar method.

### Phage thermal and pH stability assays

The thermal and pH stability of the phages were evaluated as previously described [24]. For thermal stability, 1 mL aliquots of phage lysate (approximately 10^9^ PFU/mL) were incubated at various temperatures (4, 25, 37, 40, 50, 60, 70, and 80 °C) for 1 h. For pH stability, 100 µL of phage lysate was mixed with 900 µL of SM buffer pre-adjusted to specific pH values (ranging from 2 to 14) and incubated at 37 °C for 1 h. After each treatment, the samples were serially diluted, and the viable phage titer was quantified using the double-layer agar plate method.

### Assessment of phage lytic activity at different MOI

The lytic activity of phage GSP006 against *Salmonella* Pullorum SaP001 was assessed in vitro at various MOIs. Briefly, the bacterium was cultured to the logarithmic growth phase and adjusted to a concentration of approximately 10^8^ CFU/mL. Then, 100 µL of the bacterial suspension was mixed with 100 µL of phage GSP006 suspension in a 96-well plate to achieve final MOIs of 10, 1, 0.1, 0.01, 0.001, and 0.0001. The plate was incubated at 37 °C for 12 h with continuous shaking in a Feyond-A300 microplate reader (ALLSHENG, Hangzhou, China). The optical density at 600 nm (OD_600_) was automatically measured every 30 min throughout the incubation period. The positive control consisted of the bacterial culture without phage, and the negative control contained the phage suspension in sterile LB broth.

### Genome analysis and phylogenetic analysis

To remove contaminating nucleic acids, the concentrated phage was treated with DNase I and RNase A. Subsequently, the genomic DNA of phage GSP006 was extracted using a viral genome extraction kit (Omega Bio-Tek Inc., Doraville, GA, USA). Whole-genome sequencing was performed on the Illumina MiSeq platform by Novogene Bioinformatics Technology Co., Ltd. (Beijing, China). The resulting sequencing reads were assembled *de novo* using SPAdes v3.15.2 [25]. The phage genome sequences were annotated using RAST (http://rast.nmpdr.org/) and manually verified using BLASTp (https://blast.ncbi.nlm.nih.gov/Blast.cgi). The circular genome map of GSP006 was constructed and visualized using the Proksee server (https://proksee.ca/) [26]. For phylogenetic analysis, the terminase large subunit sequences of reference phages, as recommended by the International Committee on Taxonomy of Viruses (ICTV), were retrieved from the NCBI database. A phylogenetic tree was constructed using the Neighbor-Joining method in MEGA 11 software and further optimized based on these sequences [27]. The genomic sequence similarities between phage GSP006 and other sequenced phages were analyzed using the VIRIDIC tool (https://viridic.icbm.de), with a species demarcation threshold set at 95%. Comparative genomic visualization was generated using Easyfig software [28]. The presence of potential virulence and antibiotic resistance genes in the phage genome was examined using Virulence Finder 2.0 (https://www.mgc.ac.cn/VFs/main.htm, 12 October 2025) [29] and the CARD database (https://card.mcmaster.ca/, 12 October 2025) [30] with a threshold of > 80% coverage and > 90% identity. All predicted open reading frames (ORFs) and their corresponding annotations are provided in Tables S3. The annotated phage genome was used to manually verify whether there were lysogen-associated proteins in the phage genome.

### Bactericidal effect of phage in poultry feed and drinking water

The poultry feed (Laying-hen Feed 524, Zhengda Feed Co., Ltd, Daqing, China) and drinking water (tap water) were sterilized by autoclaving. The surfaces of the poultry feed were contaminated with *Salmonella* Pullorum SaP001 at a concentration of 10⁶ CFU/mL, and the bacterium was also added directly to the drinking water. Subsequently, the samples were treated with phage at concentrations of 10⁸, 10⁹, and 10¹⁰ PFU/mL, corresponding to MOI of 100, 1,000, and 10,000, respectively. All samples were incubated at room temperature, with a control group left untreated. After storage periods of 2, 4, 6, 12, and 24 h, samples were serially diluted, plated onto LB agar plates, and incubated overnight at 37 °C for enumeration of viable bacteria.

### Data analysis

Each experiment was repeated three times, and data are presented as mean ± SD. Statistical analysis was performed using GraphPad Prism 9.0 (GraphPad Software, San Diego, CA, USA). *P* < 0.05 was considered statistically significant. ( *P < 0.05; **P < 0.01; ***P < 0.001)

## Results

### Determination of host range

**Fig. 1:**
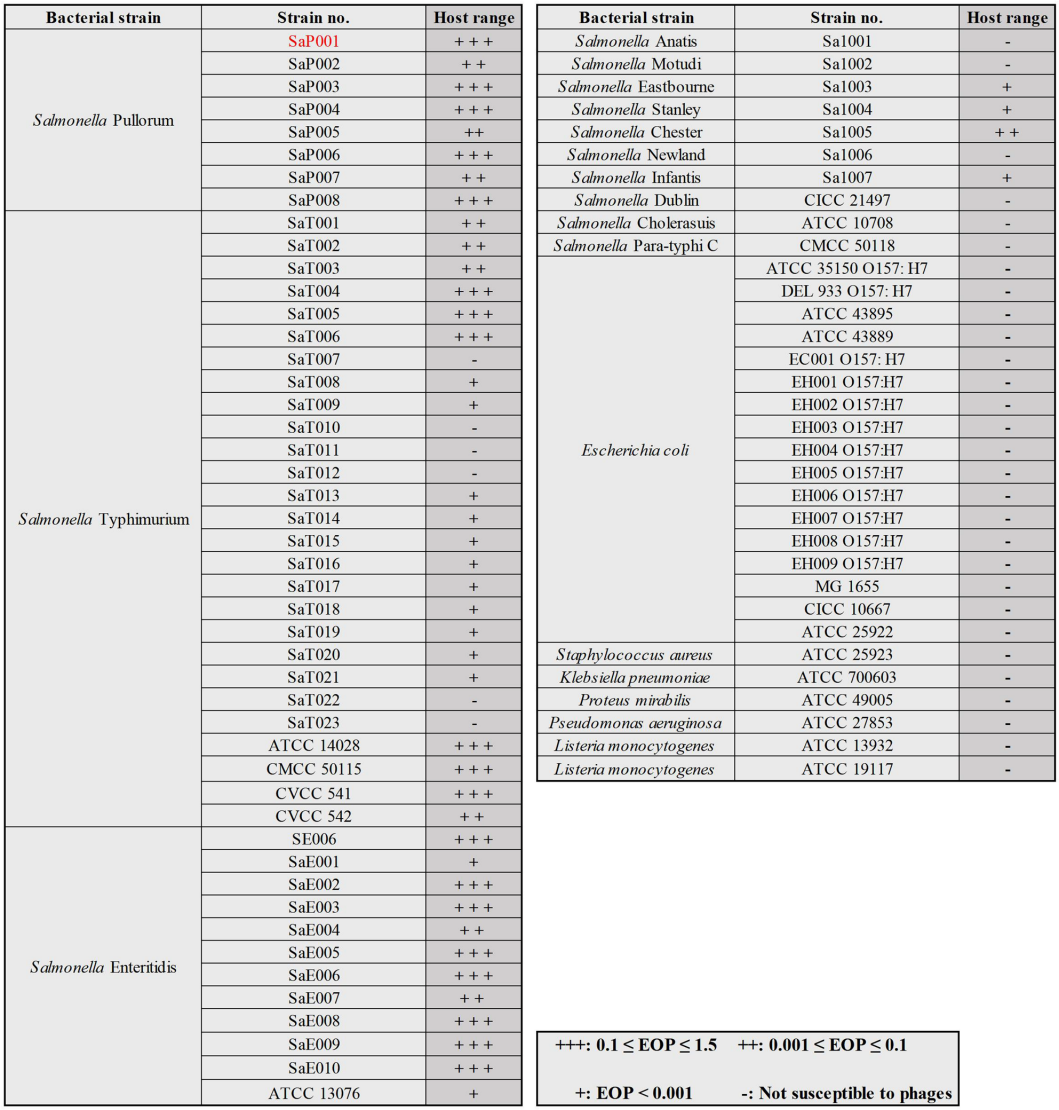
Host range analysis of phage GSP006. Host range was determined by the efficiency of plating (EOP). Lytic activity was categorized as follows: +++ (0.1 ≤ EOP ≤ 1.5), indicating strong lysis with clear plaques; ++ (0.001 ≤ EOP < 0.1), representing moderate lysis with relatively clear plaques; + (EOP < 0.001), indicating weak lysis with faint plaques; and –, no detectable lytic activity. ATCC, American Type Culture Collection; CICC, China Industrial Culture Collection; CMCC, National Center for Medical Culture Collections

The host range of phage GSP006 was determined using 57 *Salmonella* strains (Fig. 1). Phage GSP006 was capable of infecting 45 of 57 *Salmonella* strains tested. The susceptible strains included major serovars such as *Salmonella* Pullorum, *Salmonella* Enteritidis and *Salmonella* Typhimurium, as well as several other serotypes. The EOP assay revealed variations in the lytic activity of phage GSP006 across the susceptible strains. Notably, phage GSP006 exhibited high infectivity against *Salmonella* Pullorum and *Salmonella* Enteritidis.

### Morphological analysis of phage

**Fig. 2:**
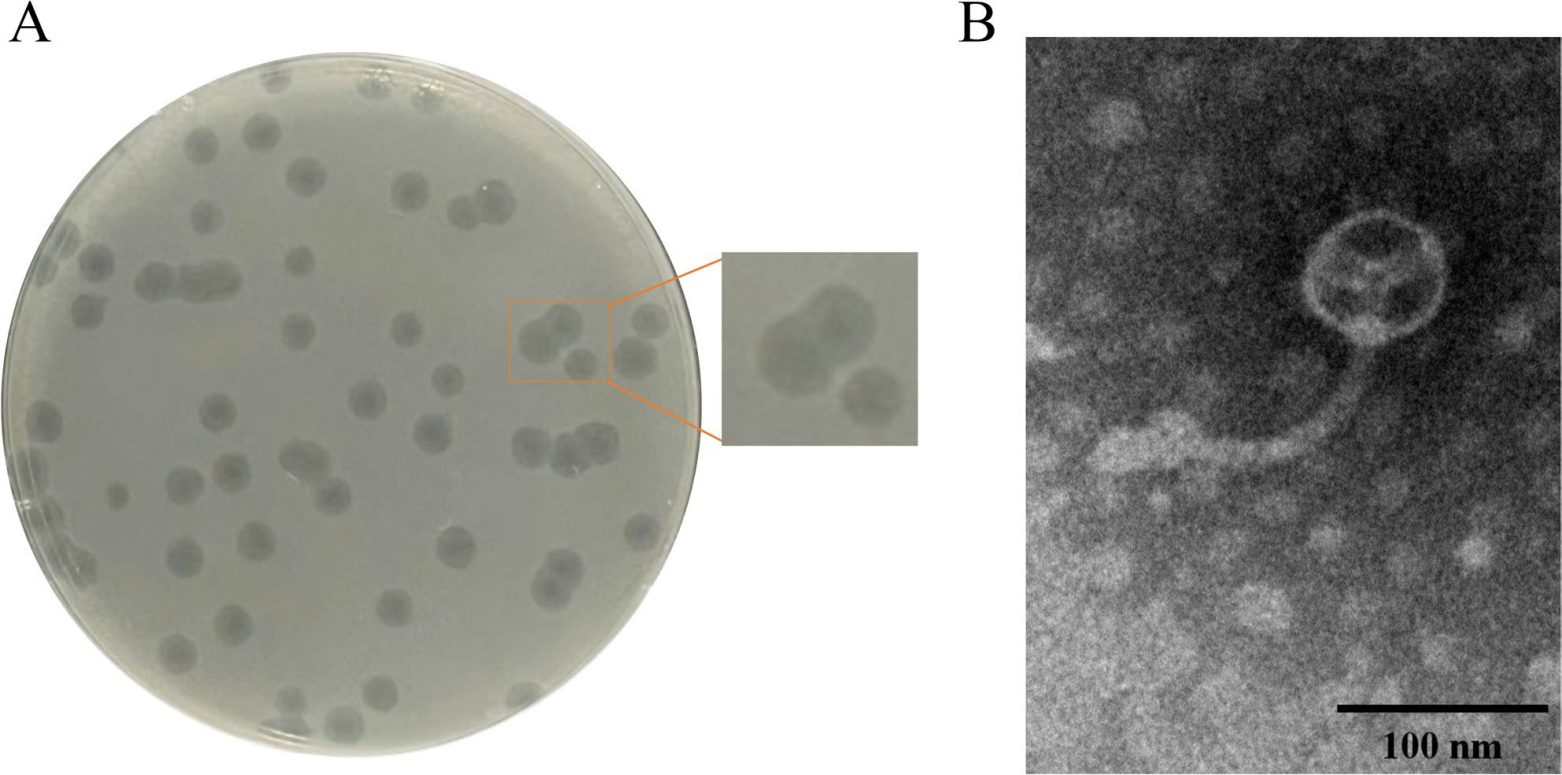
Morphology of phage GSP006. (**A**) The plaques formed by phage GSP006 on the lawns of *Salmonella* Pullorum. (**B**) TEM of phage GSP006. Scale bar, 100 nm

The plaque morphology of phage GSP006 on LB agar, following three rounds of purification, is shown in Fig. 2A. TEM revealed that phage GSP006 possesses an icosahedral head with a diameter of 64 ± 0.9 nm and a contractile tail with a length of 328 ± 1.4 nm (Fig. 2B).

### Determination of optimal multiplicity of infection

**Fig. 3:**
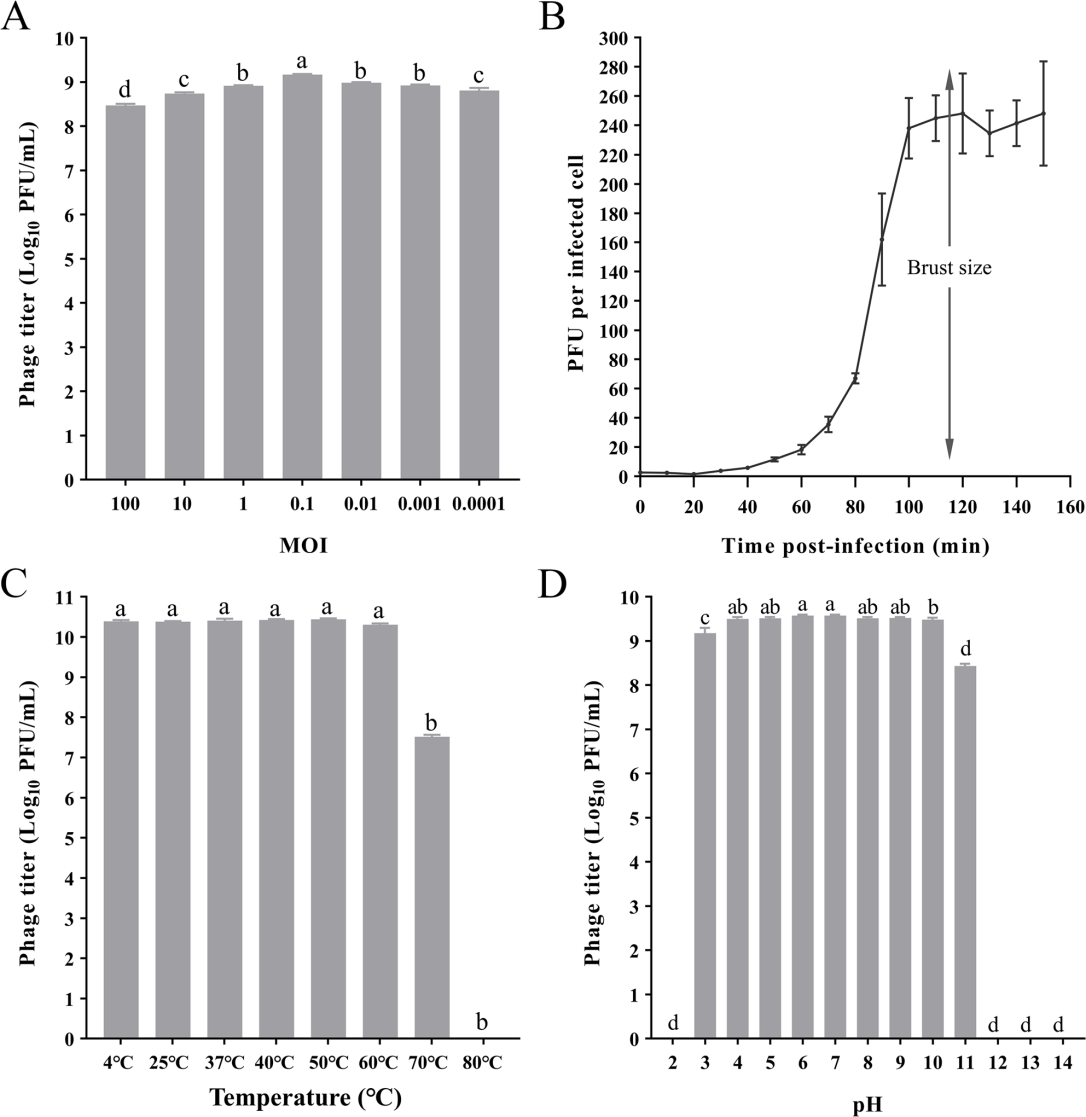
Biological characteristics of the phage GSP006 (**A**) MOI of the phage GSP006. Phage titers were determined at different MOIs values. (**B**) One-step growth curve of phage GSP006. (**C**) Thermal stability of phage GSP006. The titers of phage GSP006 were measured at different temperatures. (**D**) The pH stability of phage GSP006. The titer of phage GSP006 was determined under different pH conditions. The values represent the means and standard deviations (SD) (*n* = 3). Statistical significance was assessed by ordinary one-way ANOVA followed by Tukey’s multiple comparisons test; different letters (a, b, c, d) indicate significant differences among groups

The optimal MOI for GSP006 was determined by evaluating phage yield at various MOIs. As shown in Fig. 3A, an MOI of 0.1 resulted in the highest phage titer, and was thus identified as the optimal MOI.

### One-step growth curve

The one-step growth curve was analyzed to determine the latent period and burst size of phage GSP006. The results (Fig. 3B) revealed a short latent period of approximately 20 min, followed by a rapid lytic phase, with an average burst size of approximately 242 PFU/infected cells.

### Thermal and pH stability

In thermal stability assays, phage GSP006 maintained sustained activity across a temperature range of 4 °C to 60 °C. However, when the temperature was increased to 70 °C, its titer decreased significantly by approximately 2.86 ± 0.03 log10 PFU/mL (Fig. 3C). In pH stability tests, phage GSP006 remained stable and active within a pH range of 4 to 10, whereas its concentration dropped markedly at pH 3 and 11 (Fig. 3D).

### Assessment of the lytic activity of phage

**Fig. 4:**
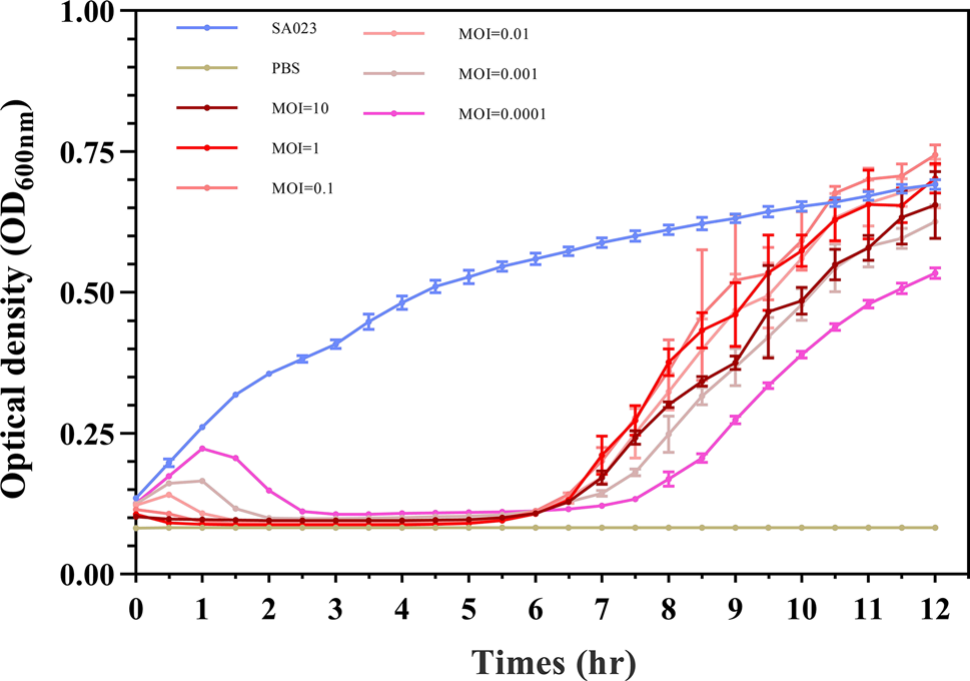
Growth curves of *Salmonella* Pullorum SaP001 in the presence of phage GSP006 at different MOIs. Bacterial growth was monitored by measuring the optical density at 600 nm (OD600) over time. The values represent the means and standard deviations (SD) (*n* = 3)

We next evaluated whether phage GSP006 could inhibit the growth of *Salmonella* Pullorum SaP001 in liquid culture. When *Salmonella* Pullorum SaP001 was infected with phage GSP006 at different multiplicities of infection (MOIs: 10, 1, 0.1, 0.01, 0.001, and 0.0001), all treatments effectively inhibited bacterial growth within 6 h compared with the positive control group. Throughout this period, the OD_600_ values of the phage-treated groups remained lower than that of the control. Moreover, higher MOIs resulted in greater reduction in OD_600_, indicating a more pronounced inhibitory effect (Fig. 4). These findings demonstrate that phage GSP006 can effectively inhibit the growth of *Salmonella* Pullorum SaP001 in liquid culture for a defined duration.

### Genomic characterization and taxonomy of GSP006

**Fig. 5:**
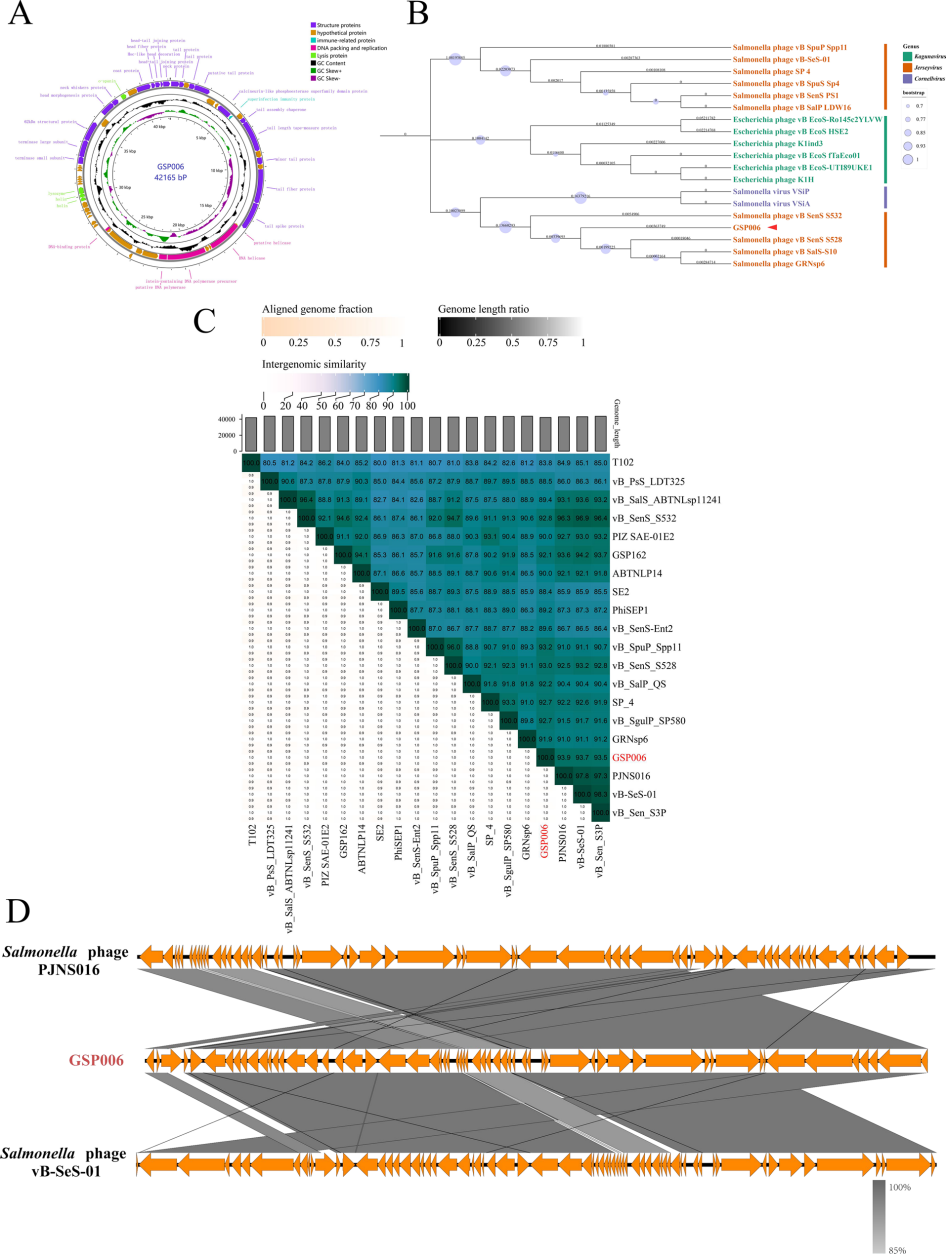
Genomic analysis of phage GSP006. (**A**) Circular genome map of phage GSP006. (B) Phylogenetic tree constructed based on the sequence of the terminase large subunit. (**C**) Heat map illustrating the whole-genome comparison of phage GSP006 with 20 other members of the Jerseyvirus genus. (**D**) Linear genome comparison of phage GSP006 with a related phage. Arrows represent ORFs and predicted proteins. Gray connecting lines indicate sequence similarity, with darker shades representing higher identity

The genome of phage GSP006 consists of double-stranded DNA, with a total length of 42,165 bp and a GC content of 50%. A total of 59 ORFs were identified through genome annotation (GenBank accession no. PV890987.1) (Fig. 5A), and their detailed information is provided in Supplementary Table S3. There were no genes associated with lysogenicity, virulence, or antibiotic resistance in the phage genome. Phylogenetic analysis based on the terminase large subunit sequence indicated that this phage should be classified under the genus *Jerseyvirus* (Fig. 5B). Consistent with this, heatmap analysis revealed that GSP006 shares nucleotide-level homology with other members of the *Jerseyvirus* genus (Fig. 5C), a finding further supported by BLASTn analysis. Further genomic alignment showed that GSP006 exhibits high similarity to phages PJNS016 (96% coverage) and vB-SeS-01 (96% coverage) (Fig. 5D), suggesting a close phylogenetic relationship among them. These results indicate that phage GSP006 is a new virulent phage belonging to the genus *Jerseyvirus*, and can be safely used for biocontrol or therapeutic applications from the genomic point of view.

### Phage GSP006 reduced Salmonella pullorum colonization in poultry feed and drinking water

**Fig. 6:**
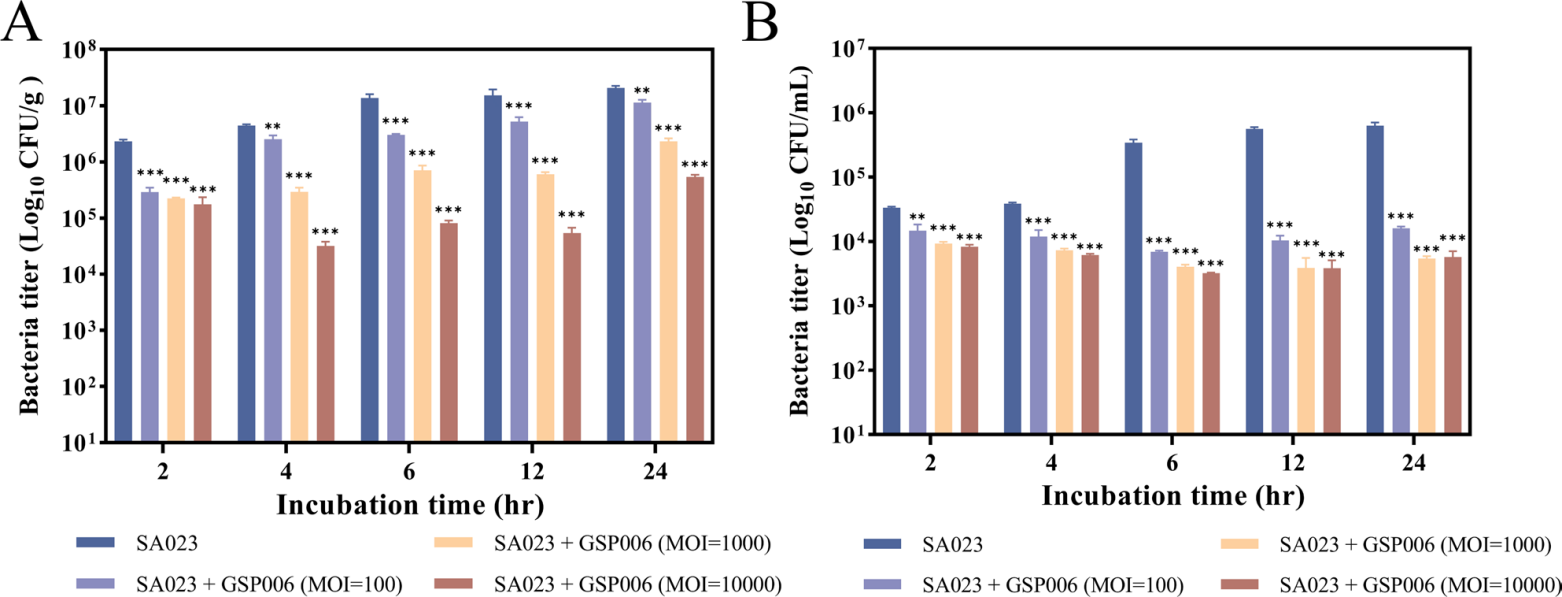
Application of phage GSP006 for biological control of *Salmonella* Pullorum SaP001. (**A**) Effect of phage on the viability of *Salmonella* Pullorum in poultry feed. (**B**) Effect of phage on the viability of *Salmonella* Pullorum in drinking water. Viable bacterial counts (CFU) following treatment with phage GSP006 were determined at the indicated time points.The values represent the means and standard deviations (SD) (*n* = 3). *P < 0.05; **P < 0.01; ***P < 0.001

To evaluate the ability of phage GSP006 to control the horizontal transmission of *Salmonella* in the poultry rearing environment, we investigated the effect of phage GSP006 on the control of *Salmonella* Pullorum SaP001 in artificially contaminated poultry feed and drinking water. When *Salmonella* Pullorum SaP001 and phage GSP006 were added to poultry feed and drinking water at a MOI of 10,000 and incubated for 24 h at 25 °C, the bacterial titers decreased by 1.9 log CFU/g in poultry feed (Fig. 6A) and by 2 log CFU/mL in drinking water (Fig. 6B). Treatment with phage at an MOI of 1,000 also significantly reduced bacterial growth, resulting in a reduction of 0.9 log CFU/g in poultry feed (Fig. 6A) and 2 log CFU/mL in drinking water (Fig. 6B) after 24 h at 25 °C. Similarly, at an MOI of 100, the bacterial titer decreased by 0.2 log CFU/g in poultry feed (Fig. 6A) and by 1.6 log CFU/mL in drinking water (Fig. 6B) under the same conditions. These results indicate that phage GSP006 has substantial antimicrobial efficacy and could be used to reduce the transmission of *Salmonella* Pullorum via poultry feed and drinking water in poultry rearing environments.

## Discussion

The history of phage therapy can be traced back to the early 20th century, when Frederick Twort and Félix d’Hérelle respectively discovered phages and proposed the possibility of their therapeutic applications [31]. Despite the impressive achievements of phage therapy in the 1920s and 1930s, the advent of antibiotics led to the decline of phage therapy in mainstream medicine. Given the escalating global crisis of antibiotic resistance, phage therapy has regained widespread attention and has shown unique advantages in the fight against drug-resistant bacteria [32–34].

In this study, we isolated a broad-host-range *Salmonella* phage from poultry farm wastewater and designated it GSP006. Phage GSP006 lyses seven serotypes of *Salmonella*, including *Salmonella* Pullorum, *Salmonella* Enteritidis, *Salmonella* Typhimurium, *Salmonella* Stanley, *Salmonella* Chester, *Salmonella* Infantis and *Salmonella* Eastbourne. The MOI value represents the ratio of phage to host bacteria. An optimal MOI can yield the highest phage titer, making its determination crucial for large-scale preparation to reduce production costs [35]. The optimal MOI for phage GSP006 was identified as 0.1 (Fig. 3A), suggesting its potential for application at high doses. According to previous studies, phages with short latency periods can lyse more bacterial cells within a given time frame [36]. The latency period of phage GSP006 was only 20 min (Fig. 3B), indicating its potential for rapid bactericidal activity. Biological characteristics of phages are key determinants of their practical applications [37]. Environmental factors such as pH and temperature significantly affect phage activity and stability. Therefore, it is essential to evaluate these parameters to define suitable conditions for phage application. Temperature tolerance experiments demonstrated that phage GSP006 maintained stable activity after treatment at temperatures ranging from 4 °C to 60 °C for 1 h; when the temperature increased to 70 °C, a portion of the phages still remained viable (Fig. 3C). In comparison with other reported phages [38, 39], GSP006 demonstrated superior tolerance to extreme temperatures. In poultry production, feed and drinking water pass through the acidic environment of the stomach. If a phage cannot survive low pH, it will fail to inhibit bacterial growth effectively after ingestion. Thus, assessing the pH stability of the phage is necessary. Phage GSP006 maintained activity within a pH range of 3 to 11 for more than 1 h (Fig. 3D), showing pH stability comparable to that of other previously reported phages [40, 41]. Collectively, the temperature and pH stability tests demonstrate that phage GSP006 can endure extreme environmental conditions, which represents an important evaluation metric and a promising attribute for its future applications.

Genome sequencing analysis revealed that phage GSP006 contains clearly defined functional modules responsible for DNA replication and modification, structural components, DNA packaging, and host lysis, which is consistent with the genomic organization observed in other *Salmonella* phages [42]. Further genomic comparisons indicated that GSP006 shares the closest phylogenetic relationship with phages PJNS016 and vB-SeS-01, whose characteristics were unknown. Despite their isolation from distinct geographical regions, the high degree of genomic homology among these phages may reflect evolutionary adaptations to a common host. Phage GSP006 exhibits a host range similar to that of the previously reported *Jerseyvirus* genus phage CKT1 [43], which may be attributed to similarities in their tail proteins. Genomic annotation identified several proteins (ORFs 27, 28, 29; Table S3) putatively involved in host lysis, such as holins and lysin, which are known to play key roles in the phage-mediated lysis of bacterial cells [44]. The absence of lysogeny-related genes in the genome confirms that GSP006 is a virulent phage. Since phages can potentially harbor and transmit hazardous genes, such as those encoding toxins, virulence factors, or antimicrobial resistance [45], rigorous screening is essential prior to their practical application to exclude candidates carrying such genetic elements. Genomic analysis of GSP006 revealed the absence of the aforementioned hazardous genes, thereby demonstrating that this phage meets safety standards for application and exhibits promising development potential.

Previous studies have demonstrated that phages can inhibit bacteria in the gut or treat *Salmonella* infections [43, 46]. Poultry can be infected with *Salmonella* in a variety of ways, including feed, water, equipment, insects, and feeders [47]. Therefore, the supplementation of phages in feed or drinking water at the source may reduce the environmental load of *Salmonella*, thereby decreasing horizontal transmission and exerting a preventive effect. In this study, phage GSP006 was applied by spraying to artificially contaminated chicken feed and drinking water inoculated with *Salmonella* Pullorum. The results demonstrated a significant reduction in *Salmonella* concentration in the treated groups (Fig. 6A, B). This finding is consistent with the study by Gao et al., in which phage GSP044 also significantly reduced artificially contaminated *Salmonella* in poultry feed and drinking water [12]. Furthermore, Pourabadeh et al. reported that a BP phage cocktail significantly reduced colonization of *Salmonella* Enteritidis and *Salmonella* Typhimurium in multiple organs of broilers [48]. Similarly, Nabil et al. found that prophylactic administration of a phage cocktail via drinking water effectively protected birds against *Salmonella*, whereas non-pretreated groups developed diarrhea and organ lesions [49]. Phages have also shown significant inhibitory effects against *Salmonella* in various food matrices, such as fruit juice [50], milk [51], duck meat [52], beef [53], lettuce [51], and eggs [24], although complete eradication of the host bacteria was not achieved in any of these studies. Although the present study confirms the efficacy of phage GSP006 in controlling *Salmonella* in feed and water under laboratory conditions, these represent an idealized environment rather than actual farm conditions, and practical applications still face numerous constraints. Therefore, future large-scale field trials are warranted to evaluate the effectiveness of this approach in controlling the transmission of avian *Salmonella* in real-world environments.

## Conclusion

In this study, we isolated a broad-host-range *Salmonella* phage GSP006 and further characterized GSP006 using *Salmonella* Pullorum SaP001 as the host bacterium. Biological characterization and genomic analysis showed that phage GSP006 has excellent tolerance range (temperature: 4 to 60 °C; pH: 3 to 11), lysis ability and biological safety. In addition, phage GSP006 showed significant effects in reducing *Salmonella* Pullorum in poultry feed and drinking water. Although phage GSP006 as an eco-friendly biocontrol agent is expected to be an antibiotic alternative antimicrobial agent, there is still a lot of work to be done to realize the application of GSP006 for preventing and controlling *Salmonella* infections in poultry.

## Supplementary Information

Below is the link to the electronic supplementary material.

Supplementary Material 1

Supplementary Material 2

Supplementary Material 3

## Data Availability

The datasets used and/or analysed during the current study are available from the corresponding author on reasonable request.
