# Supplementary material for: Characterization of broad-host-range Salmonella phage GSP006 and its efficacy in controlling Salmonella Pullorum contamination in poultry feed and drinking water
Source: BMC Biotechnol. 2026 Jan 15;26:19. doi: 10.1186/s12896-026-01100-w (PMC12892657; doi:10.1186/s12896-026-01100-w)
Supplement: Supplementary file 1 — Supplementary Material 1 [file 12896_2026_1100_MOESM1_ESM.docx]

**Characterization of Broad-Host-Range *Salmonella* Phage GSP006 and Its Efficacy in Controlling *Salmonella* Pullorum Contamination in Poultry Feed and Drinking Water**

Shenyu Pang^1^, Hongyang Zhang^1^, Xincong Liu^1^, Jilai Wang^1^, Shunyuan Pan^1^, Xiangyu Kong^1^, Jun Song^1, 2, 3, *^ Dongyang Gao^1, 2, 3, *^

**Supplementary Information**

**Supplementary Tables 1, 2.**

**Supplementary Tables**

**Table S1 The *Salmonella* strains used in this study.**

| **Strain** | **Strain ID number** | **Source of strains** |
| --- | --- | --- |
| *Salmonella enterica* serovar Pullorum (*S.* Pullorum) | SaP001 | chicken, Lab Stock |
|  | SaP002 | chicken, Lab Stock |
|  | SaP003 | chicken, Lab Stock |
|  | SaP004 | chicken, Lab Stock |
|  | SaP005 | chicken, Lab Stock |
|  | SaP006 | chicken, Lab Stock |
|  | SaP007 | chicken, Lab Stock |
|  | SaP008 | chicken, Lab Stock |
| *Salmonella enterica* serovar Typhimurium (*S.* Typhimurium) | SaT001 | chicken, Lab Stock |
|  | SaT002 | chicken, Lab Stock |
|  | SaT003 | chicken, Lab Stock |
|  | SaT004 | chicken, Lab Stock |
|  | SaT005 | chicken, Lab Stock |
|  | SaT006 | chicken, Lab Stock |
|  | SaT007 | chicken, Lab Stock |
|  | SaT008 | chicken, Lab Stock |
|  | SaT009 | pig, Lab Stock |
|  | SaT010 | pig, Lab Stock |
|  | SaT011 | pig, Lab Stock |
|  | SaT012 | pig, Lab Stock |
|  | SaT013 | pig, Lab Stock |
|  | SaT014 | pig, Lab Stock |
|  | SaT015 | pig, Lab Stock |
|  | SaT016 | pig, Lab Stock |
|  | SaT017 | pig, Lab Stock |
|  | SaT018 | pig, Lab Stock |
|  | SaT019 | pig, Lab Stock |
|  | SaT020 | pig, Lab Stock |
|  | SaT021 | pig, Lab Stock |
|  | SaT022 | pig, Lab Stock |
|  | SaT023 | pig, Lab Stock |
|  | ATCC 14028 | ATCC, Lab Stock |
|  | CMCC 50115 | CMCC,Lab Stock |
|  | CVCC 541 | CVCC, Lab Stock |
|  | CVCC 542 | CVCC, Lab Stock |
| *Salmonella enterica* serovar [Enteritidis](https://www.ncbi.nlm.nih.gov/nuccore/CP082726.1) (*S.* Enteritidis) | SE006 | [1]，Lab Stock |
|  | SaE001 | chicken, Lab Stock |
|  | SaE002 | chicken, Lab Stock |
|  | SaE003 | chicken, Lab Stock |
|  | SaE004 | chicken, Lab Stock |
|  | SaE005 | chicken, Lab Stock |
|  | SaE006 | chicken, Lab Stock |
|  | SaE007 | chicken, Lab Stock |
|  | SaE008 | chicken, Lab Stock |
|  | SaE009 | chicken, Lab Stock |
|  | SaE0010 | chicken, Lab Stock |
|  | ATCC 13076 | ATCC, Lab Stock |
| *Salmonella enterica* serovar Anatis (*S.* Anatis) | Sal001 | pork, Lab Stock |
| *Salmonella enterica* serovar Motudi (*S.* Motudi) | Sal002 | pork, Lab Stock |
| *Salmonella enterica* serovar Eastbourne (*S.* Eastbourne) | Sal003 | pork, Lab Stock |
| *Salmonella enterica* serovar Stanley (*S.* Stanley) | Sal004 | pork, Lab Stock |
| *Salmonella enterica* serovar Chester (*S.* Chester) | Sal005 | pork, Lab Stock |
| *Salmonella enterica* serovar Newland (*S.* Newland) | Sal006 | pork, Lab Stock |
| *Salmonella enterica* serovar Infantis (*S.* Infantis) | Sal007 | chicken meat, Lab Stock |
| *Salmonella enterica* serovar Dublin (*S.* Dublin) | CICC 21497 | CICC, Lab Stock |
| *Salmonella enterica* serovar Cholerasuist (*S.* Cholerasuist) | ATCC 10708 | ATCC, Lab Stock |
| *Salmonella enterica* serovar Para-typhi C (*S.* Para-typhi C) | CMCC 50118 | CMCC , Lab Stock |

ATCC: American Type Culture Collection; CMCC: National Center for Medical Culture Collections; CICC: China Center of Industrial Culture Collection; CVCC: National Center for Veterinary Culture Collection; NCTC: National Counterterrorism Center.

**Table S2 The *Escherichia coli* and other strains used in this study.**

| **Strain** | **Strain ID number** | **Source of strains** |
| --- | --- | --- |
| *Escherichia coli* (*E. coli*) | ATCC 35150 O157:H7 | ATCC, Lab Stock |
|  | EDL 933 O157:H7 | Lab Stock |
|  | ATCC 43895 | ATCC, Lab Stock |
|  | ATCC 43889 | ATCC, Lab Stock |
|  | EC001 O157:H7 | Lab Stock |
|  | EH001 O157:H7 | Lab Stock |
|  | EH002 O157:H7 | Lab Stock |
|  | EH003 O157:H7 | Lab Stock |
|  | EH004 O157:H7 | Lab Stock |
|  | EH005 O157:H7 | Lab Stock |
|  | EH006 O157:H7 | Lab Stock |
|  | EH007 O157:H7 | Lab Stock |
|  | EH008 O157:H7 | Lab Stock |
|  | EH009 O157:H7 | Lab Stock |
|  | MG 1655 | Lab Stock |
|  | CICC 10667 | CICC, Lab Stock |
|  | ATCC 25922 | ATCC, Lab Stock |
| *Staphylococcus aureus* | ATCC 25923 | ATCC, Lab Stock |
| *Klebsiella pneumoniae* | ATCC 700603 | ATCC, Lab Stock |
| *Proteus mirabilis* | CMCC 49005 | CMCC, Lab Stock |
| *Pseudomonas aeruginosa* | ATCC 27853 | ATCC, Lab Stock |
| *Listeria monocytogenes* | ATCC 13932 | ATCC, Lab Stock |
| *Listeria monocytogenes* | ATCC 19117 | ATCC, Lab Stock |

ATCC: American Type Culture Collection; CMCC: National Center for Medical Culture Collections; CICC: China Center of Industrial Culture Collection; CVCC: National Center for Veterinary Culture Collection; NCTC: National Counterterrorism Center.

**References**

1. Gao D, Ji H, Wang L, Li X, Hu D, Zhao J, et al. Fitness Trade-Offs in Phage Cocktail-Resistant *Salmonella* enterica Serovar Enteritidis Results in Increased Antibiotic Susceptibility and Reduced Virulence. Microbiol Spectr. 2022;10(5):e0291422; doi: 10.1128/spectrum.02914-22.
